# 2-[(*Z*)-1,1-Dioxo-2-(2,4,5-tri­fluoro­benz­yl)-3,4-di­hydro-2*H*-1,2-benzo­thia­zin-4-yl­idene]acetic acid

**DOI:** 10.1107/S1600536814008903

**Published:** 2014-05-03

**Authors:** Shagufta Parveen, Saghir Hussain, Shaojuan Zhu, Xin Hao, Changjin Zhu

**Affiliations:** a School of Chemical Engineering and Environment, Beijing Institute of Technology, Beijing 100081, People’s Republic of China

## Abstract

In the title compound, C_17_H_12_F_3_NO_4_S, the heterocyclic thia­zine ring adopts a half-chair conformation and the dihedral angle between the benzene rings is 43.28 (9)°. The α,β-unsaturated C=C group is inclined at an angle of 21.0 (3)° to the benzene ring of the benzo­thia­zine moiety. In the crystal, inversion dimers linked by pairs of carb­oxy­lic acid O—H⋯O hydrogen bonds generate *R*_2_^2^(8) loops. Each of the F atoms accepts a C_a_—H⋯F (a = aromatic) hydrogen bond from an adjacent mol­ecule, resulting in (001) sheets.

## Related literature   

For pharmaceuticals properties of 1,2-benzo­thia­zines, see: Lombardino *et al.* (1971 ▶); Turck *et al.* (1996 ▶); Zia-ur-Rehman *et al.* (2005 ▶). For the biological properties and synthetic details of the title compound, see: Parveen *et al.* (2014 ▶). For related structures, see: Ahmad *et al.* (2008 ▶); Zia-ur-Rehman *et al.* (2008 ▶); Yang *et al.* (2012 ▶). For graph-set analysis, see: Etter *et al.* (1990 ▶).
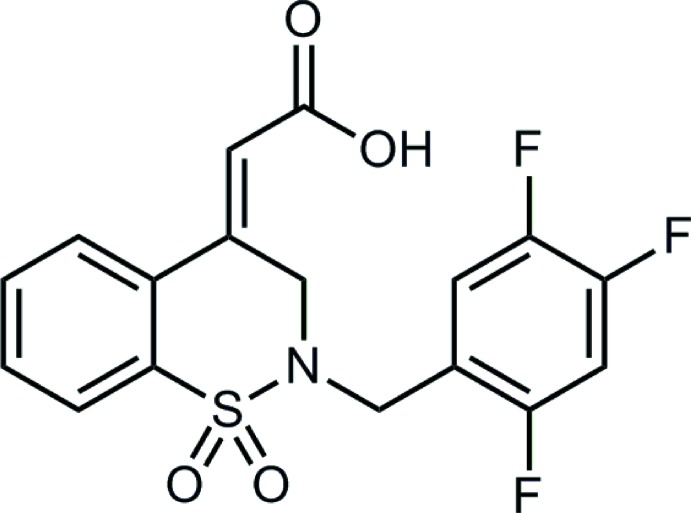


## Experimental   

### 

#### Crystal data  

C_17_H_12_F_3_NO_4_S
*M* *_r_* = 383.34
Monoclinic, 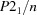

*a* = 6.6085 (12) Å
*b* = 12.649 (3) Å
*c* = 18.757 (4) Å
β = 99.601 (2)°
*V* = 1545.9 (5) Å^3^
*Z* = 4
Mo *K*α radiation
μ = 0.27 mm^−1^
*T* = 153 K
0.31 × 0.21 × 0.07 mm

#### Data collection  

Rigaku AFC10/Saturn724+ CCD-detector diffractometer
Absorption correction: multi-scan (*CrystalClear*; Rigaku, 2008 ▶) *T*_min_ = 0.910, *T*_max_ = 0.970
13553 measured reflections
4123 independent reflections
3594 reflections with *I* > 2σ(*I*)
*R*_int_ = 0.033

#### Refinement  

*R*[*F* ^2^ > 2σ(*F* ^2^)] = 0.049
*wR*(*F* ^2^) = 0.130
*S* = 1.00
4123 reflections
239 parameters
H atoms treated by a mixture of independent and constrained refinement
Δρ_max_ = 0.30 e Å^−3^
Δρ_min_ = −0.46 e Å^−3^

### 

Data collection: *CrystalClear* (Rigaku, 2008 ▶); cell refinement: *CrystalClear*; data reduction: *CrystalClear*; program(s) used to solve structure: *SHELXS97* (Sheldrick, 2008 ▶); program(s) used to refine structure: *SHELXL97* (Sheldrick, 2008 ▶); molecular graphics: *DIAMOND* (Brandenburg, 1998 ▶); software used to prepare material for publication: *CrystalStructure* (Rigaku, 2008 ▶).

## Supplementary Material

Crystal structure: contains datablock(s) I, New_Global_Publ_Block. DOI: 10.1107/S1600536814008903/zs2294sup1.cif

Structure factors: contains datablock(s) I. DOI: 10.1107/S1600536814008903/zs2294Isup2.hkl

Supporting information file. DOI: 10.1107/S1600536814008903/zs2294Isup3.mol

Supporting information file. DOI: 10.1107/S1600536814008903/zs2294Isup4.cml

CCDC reference: 998389

Additional supporting information:  crystallographic information; 3D view; checkCIF report

## Figures and Tables

**Table 1:** Hydrogen-bond geometry (Å, °)

*D*—H⋯*A*	*D*—H	H⋯*A*	*D*⋯*A*	*D*—H⋯*A*
O3—H3*O*⋯O4^i^	0.95 (3)	1.71 (3)	2.6454 (19)	170 (3)
C12—H12⋯F3^ii^	0.95	2.50	3.448 (2)	178
C15—H15⋯F1^iii^	0.95	2.48	3.430 (2)	179
C5—H5⋯F2^iv^	0.95	2.49	3.269 (2)	140

Symmetry codes: (i) 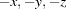; (ii) 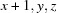; (iii) 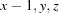; (iv) 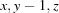.

**Fig. 1.**
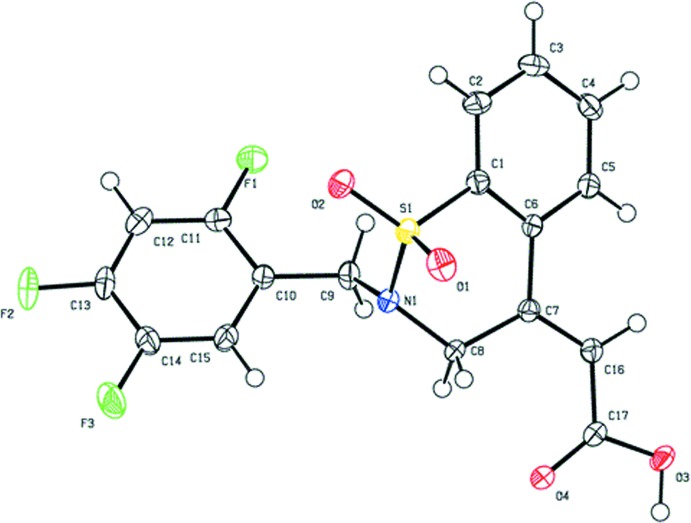
The molecular structure of the title compound with the atom numbering scheme. Displacement ellipsoids are drawn at the 30% probability level.

**Fig. 2.**
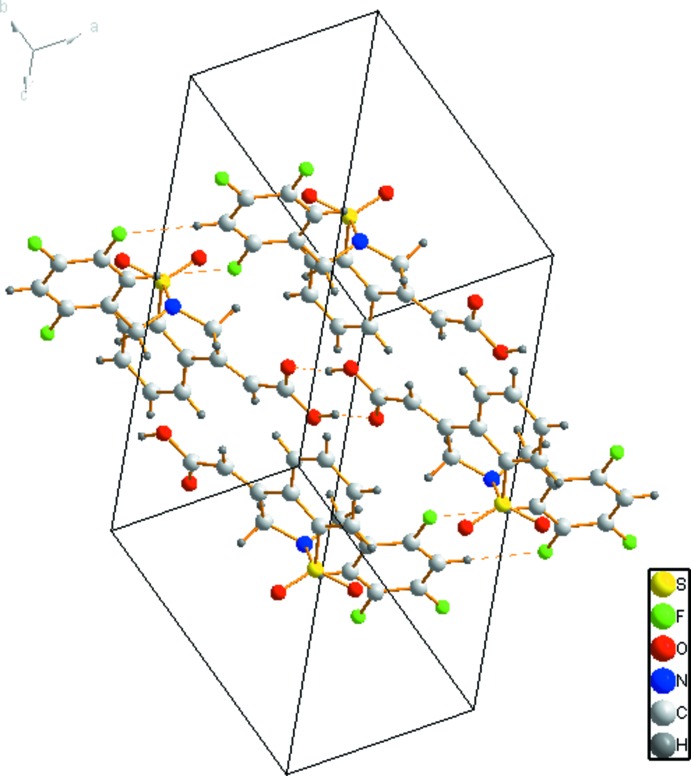
A view of the O—H···O and C—H···F hydrogen-bonding interactions (dotted lines) in the crystal structure of the title compound. H atoms non-participating in hydrogen-bonding are omitted for clarity.

## supplementary crystallographic information

### 1. Comment

Benzothiazine derivatives have been found to posses versatile biological activities such as anti-inflammatory, antioxidant and anti-bacterial (Lombardino *et al.*, 1971; Zia-ur-Rehman *et al.*, 2005). Derivatives of 1,2-benzothiazine-1,1-dioxide also reported as aldose reductase inhibitors (Parveen *et al.*, 2014). We report here the structure of the title compound, C_17_H_12_F_3_NO_4_S, as an extension of this study. In this compound, (Fig. 1) the heterocyclic thiazine ring adopts a half chair conformation and the dihedral angle between the two benzene rings is 43.28 (9)°. The α,β-unsaturated C═C is inclined at an angle of 21.0 (3)° (torsion angle C5—C6—C7—C16) to the mean plane of the benzene ring (C1–C6). In the crystal, the molecules form centrosymmetric cyclic dimers through duplex intermolecular carboxylic acid O—H···O hydrogen bonds [graph set *R*^2^_2_(8) (Etter *et al.*, 1990)] (Table 1) while all of the fluorine atoms on the benzyl ring are involved in intermolecular aromatic C—H···F hydrogen-bonding interactions, giving a two-dimensional network structure lying parallel to (001) (Fig. 3).

### 2. Experimental

A mixture of *Z*-2-[2-(2,4,5-trifluorobenzyl)-1,1-dioxido-2*H*-1,2-benzothiazin- 4(3*H*)-ylidene]acetic acid methyl ester (0.5 mmol), 1,4-dioxane (5 mL) and 10*M* hydrochloric acid (8 mL) was refluxed at 80°C for 12 h. The precipitate formed was then filtered and washed with cold water. The crude product was purified by flash chromatography. Crystals suitable for X-ray crystallography were obtained by slow evaporation of a solution of the title compound in ethanol (yield = 70%).

### 3. Refinement

The H atom bonded to O1 was located from a difference-Fourier map and refined freely. The remaining H atoms were positioned geometrically, with C—H = 0.95 and 0.99 Å for aromatic and methylene H, respectively, and constrained to ride on their parent atoms with *U*_iso_(H) = 1.2*U*_eq_(C).

### Crystal data

**Table:**

C_17_H_12_F_3_NO_4_S	*F*(000) = 784
*M**_r_* = 383.34	*D*_x_ = 1.647 Mg m^−^^3^
Monoclinic, *P*2_1_/*n*	Mo *K*α radiation, λ = 0.71073 Å
*a* = 6.6085 (12) Å	Cell parameters from 5016 reflections
*b* = 12.649 (3) Å	θ = 2.2–29.1°
*c* = 18.757 (4) Å	µ = 0.27 mm^−^^1^
β = 99.601 (2)°	*T* = 153 K
*V* = 1545.9 (5) Å^3^	Prism, colorless
*Z* = 4	0.31 × 0.21 × 0.07 mm

### Data collection

**Table:**

Rigaku AFC10/Saturn724+ CCD-detector diffractometer	4123 independent reflections
Radiation source: Rotating Anode	3594 reflections with *I* > 2σ(*I*)
Graphite monochromator	*R*_int_ = 0.033
Detector resolution: 28.5714 pixels mm^-1^	θ_max_ = 29.1°, θ_min_ = 3.1°
φ and ω scans	*h* = −7→9
Absorption correction: multi-scan (*CrystalClear*; Rigaku, 2008)	*k* = −17→17
*T*_min_ = 0.910, *T*_max_ = 0.970	*l* = −24→25
13553 measured reflections	

### Refinement

**Table:**

Refinement on *F*^2^	Primary atom site location: structure-invariant direct methods
Least-squares matrix: full	Secondary atom site location: difference Fourier map
*R*[*F*^2^ > 2σ(*F*^2^)] = 0.049	Hydrogen site location: inferred from neighbouring sites
*wR*(*F*^2^) = 0.130	H atoms treated by a mixture of independent and constrained refinement
*S* = 1.00	*w* = 1/[σ^2^(*F*_o_^2^) + (0.0691*P*)^2^ + 0.960*P*] where *P* = (*F*_o_^2^ + 2*F*_c_^2^)/3
4123 reflections	(Δ/σ)_max_ = 0.001
239 parameters	Δρ_max_ = 0.30 e Å^−^^3^
0 restraints	Δρ_min_ = −0.46 e Å^−^^3^

### Special details

**Table:**

Geometry. All esds (except the esd in the dihedral angle between two l.s. planes) are estimated using the full covariance matrix. The cell esds are taken into account individually in the estimation of esds in distances, angles and torsion angles; correlations between esds in cell parameters are only used when they are defined by crystal symmetry. An approximate (isotropic) treatment of cell esds is used for estimating esds involving l.s. planes.
Refinement. Refinement of F^2^ against ALL reflections. The weighted R-factor wR and goodness of fit S are based on F^2^, conventional R-factors R are based on F, with F set to zero for negative F^2^. The threshold expression of F^2^ > 2sigma(F^2^) is used only for calculating R-factors(gt) etc. and is not relevant to the choice of reflections for refinement. R-factors based on F^2^ are statistically about twice as large as those based on F, and R- factors based on ALL data will be even larger.

### Fractional atomic coordinates and isotropic or equivalent isotropic displacement parameters (Å^2^)

**Table:**

	*x*	*y*	*z*	*U*_iso_*/*U*_eq_	
S1	0.77960 (7)	0.24006 (4)	0.22711 (2)	0.01907 (13)	
F1	1.19907 (18)	0.38684 (10)	0.09496 (7)	0.0309 (3)	
F2	0.9333 (2)	0.73151 (9)	0.06966 (8)	0.0356 (3)	
F3	0.5560 (2)	0.65732 (11)	0.07640 (9)	0.0451 (4)	
O1	0.6212 (2)	0.21803 (12)	0.26843 (8)	0.0274 (3)	
O2	0.9255 (2)	0.32226 (11)	0.24924 (8)	0.0278 (3)	
O3	0.2021 (2)	−0.09741 (11)	0.02713 (8)	0.0225 (3)	
O4	0.1809 (2)	0.07828 (11)	0.04036 (8)	0.0236 (3)	
N1	0.6674 (2)	0.26344 (12)	0.14388 (8)	0.0179 (3)	
C1	0.9094 (3)	0.12210 (15)	0.21459 (9)	0.0184 (4)	
C2	1.1103 (3)	0.10765 (16)	0.24959 (10)	0.0231 (4)	
H2	1.1782	0.1613	0.2800	0.028*	
C3	1.2102 (3)	0.01352 (17)	0.23936 (11)	0.0237 (4)	
H3	1.3476	0.0024	0.2626	0.028*	
C4	1.1085 (3)	−0.06389 (15)	0.19517 (11)	0.0221 (4)	
H4	1.1748	−0.1295	0.1901	0.027*	
C5	0.9114 (3)	−0.04731 (15)	0.15816 (10)	0.0195 (4)	
H5	0.8469	−0.1005	0.1265	0.023*	
C6	0.8059 (3)	0.04668 (14)	0.16672 (9)	0.0156 (3)	
C7	0.5982 (3)	0.06696 (14)	0.12487 (9)	0.0161 (3)	
C8	0.5169 (3)	0.18039 (14)	0.11740 (11)	0.0193 (4)	
H8A	0.4635	0.1941	0.0657	0.023*	
H8B	0.4000	0.1863	0.1440	0.023*	
C9	0.8116 (3)	0.29315 (15)	0.09448 (11)	0.0208 (4)	
H9A	0.7563	0.2682	0.0450	0.025*	
H9B	0.9449	0.2576	0.1103	0.025*	
C10	0.8449 (3)	0.41109 (14)	0.09298 (10)	0.0182 (4)	
C11	1.0366 (3)	0.45337 (15)	0.09093 (10)	0.0195 (4)	
C12	1.0725 (3)	0.56021 (16)	0.08396 (10)	0.0230 (4)	
H12	1.2068	0.5864	0.0829	0.028*	
C13	0.9075 (3)	0.62708 (15)	0.07861 (10)	0.0236 (4)	
C14	0.7142 (3)	0.58847 (16)	0.08163 (12)	0.0262 (4)	
C15	0.6818 (3)	0.48208 (16)	0.08880 (12)	0.0252 (4)	
H15	0.5477	0.4567	0.0909	0.030*	
C16	0.4816 (3)	−0.01466 (14)	0.09595 (10)	0.0186 (4)	
H16	0.5364	−0.0838	0.1045	0.022*	
C17	0.2763 (3)	−0.00501 (15)	0.05225 (10)	0.0184 (4)	
H3O	0.064 (4)	−0.083 (2)	0.0060 (15)	0.043 (8)*	

### Atomic displacement parameters (Å^2^)

**Table:**

	*U*^11^	*U*^22^	*U*^33^	*U*^12^	*U*^13^	*U*^23^
S1	0.0204 (2)	0.0161 (2)	0.0201 (2)	−0.00048 (17)	0.00145 (17)	−0.00322 (16)
F1	0.0183 (6)	0.0291 (7)	0.0454 (8)	0.0025 (5)	0.0058 (5)	0.0017 (6)
F2	0.0519 (9)	0.0149 (6)	0.0427 (8)	−0.0093 (6)	0.0157 (6)	0.0012 (5)
F3	0.0326 (7)	0.0217 (7)	0.0823 (12)	0.0090 (6)	0.0130 (7)	0.0066 (7)
O1	0.0321 (8)	0.0254 (7)	0.0268 (7)	0.0035 (6)	0.0113 (6)	0.0000 (6)
O2	0.0283 (8)	0.0197 (7)	0.0324 (8)	−0.0045 (6)	−0.0040 (6)	−0.0075 (6)
O3	0.0186 (7)	0.0180 (6)	0.0294 (7)	−0.0040 (5)	−0.0005 (5)	−0.0066 (5)
O4	0.0183 (6)	0.0183 (6)	0.0315 (7)	−0.0028 (5)	−0.0034 (5)	0.0009 (5)
N1	0.0173 (7)	0.0143 (7)	0.0214 (7)	−0.0022 (6)	0.0010 (6)	−0.0003 (6)
C1	0.0204 (9)	0.0176 (8)	0.0167 (8)	−0.0008 (7)	0.0020 (7)	−0.0003 (6)
C2	0.0211 (9)	0.0265 (10)	0.0193 (9)	−0.0010 (8)	−0.0037 (7)	−0.0014 (7)
C3	0.0166 (9)	0.0307 (10)	0.0227 (9)	0.0035 (8)	−0.0002 (7)	0.0041 (8)
C4	0.0218 (9)	0.0185 (9)	0.0264 (9)	0.0049 (7)	0.0053 (7)	0.0044 (7)
C5	0.0194 (9)	0.0162 (8)	0.0231 (9)	−0.0016 (7)	0.0039 (7)	0.0005 (7)
C6	0.0157 (8)	0.0141 (8)	0.0173 (8)	−0.0017 (6)	0.0033 (6)	0.0014 (6)
C7	0.0158 (8)	0.0162 (8)	0.0165 (8)	−0.0013 (6)	0.0034 (6)	0.0012 (6)
C8	0.0141 (8)	0.0139 (8)	0.0283 (9)	−0.0026 (7)	−0.0016 (7)	−0.0003 (7)
C9	0.0232 (9)	0.0146 (8)	0.0257 (9)	−0.0012 (7)	0.0074 (7)	−0.0007 (7)
C10	0.0193 (9)	0.0158 (8)	0.0191 (8)	−0.0016 (7)	0.0020 (7)	0.0007 (6)
C11	0.0175 (9)	0.0219 (9)	0.0188 (8)	0.0007 (7)	0.0019 (7)	−0.0014 (7)
C12	0.0231 (9)	0.0262 (10)	0.0195 (9)	−0.0088 (8)	0.0035 (7)	−0.0021 (7)
C13	0.0352 (11)	0.0140 (8)	0.0218 (9)	−0.0066 (8)	0.0056 (8)	−0.0008 (7)
C14	0.0244 (10)	0.0178 (9)	0.0365 (11)	0.0035 (8)	0.0056 (8)	0.0022 (8)
C15	0.0179 (9)	0.0194 (9)	0.0387 (11)	−0.0026 (7)	0.0059 (8)	0.0009 (8)
C16	0.0185 (9)	0.0151 (8)	0.0220 (9)	−0.0002 (7)	0.0030 (7)	−0.0009 (7)
C17	0.0172 (8)	0.0185 (8)	0.0194 (8)	−0.0038 (7)	0.0028 (7)	−0.0011 (7)

### Geometric parameters (Å, º)

**Table:**

S1—O1	1.4302 (15)	C5—C6	1.401 (2)
S1—O2	1.4316 (14)	C5—H5	0.9500
S1—N1	1.6395 (16)	C6—C7	1.485 (2)
S1—C1	1.7562 (19)	C7—C16	1.347 (2)
F1—C11	1.356 (2)	C7—C8	1.530 (2)
F2—C13	1.346 (2)	C8—H8A	0.9900
F3—C14	1.352 (2)	C8—H8B	0.9900
O3—C17	1.323 (2)	C9—C10	1.509 (2)
O3—H3O	0.95 (3)	C9—H9A	0.9900
O4—C17	1.229 (2)	C9—H9B	0.9900
N1—C8	1.475 (2)	C10—C11	1.381 (3)
N1—C9	1.484 (2)	C10—C15	1.395 (3)
C1—C2	1.392 (3)	C11—C12	1.382 (3)
C1—C6	1.407 (2)	C12—C13	1.371 (3)
C2—C3	1.390 (3)	C12—H12	0.9500
C2—H2	0.9500	C13—C14	1.377 (3)
C3—C4	1.383 (3)	C14—C15	1.373 (3)
C3—H3	0.9500	C15—H15	0.9500
C4—C5	1.386 (3)	C16—C17	1.468 (3)
C4—H4	0.9500	C16—H16	0.9500
			
O1—S1—O2	120.19 (9)	N1—C8—H8B	108.4
O1—S1—N1	107.19 (9)	C7—C8—H8B	108.4
O2—S1—N1	108.70 (9)	H8A—C8—H8B	107.5
O1—S1—C1	108.97 (9)	N1—C9—C10	111.90 (15)
O2—S1—C1	109.60 (9)	N1—C9—H9A	109.2
N1—S1—C1	100.32 (8)	C10—C9—H9A	109.2
C17—O3—H3O	104.8 (17)	N1—C9—H9B	109.2
C8—N1—C9	115.93 (15)	C10—C9—H9B	109.2
C8—N1—S1	111.38 (12)	H9A—C9—H9B	107.9
C9—N1—S1	113.89 (12)	C11—C10—C15	116.92 (17)
C2—C1—C6	122.42 (17)	C11—C10—C9	121.38 (17)
C2—C1—S1	119.82 (14)	C15—C10—C9	121.57 (17)
C6—C1—S1	117.72 (14)	F1—C11—C10	118.64 (17)
C3—C2—C1	119.01 (18)	F1—C11—C12	117.72 (17)
C3—C2—H2	120.5	C10—C11—C12	123.64 (18)
C1—C2—H2	120.5	C13—C12—C11	117.60 (18)
C4—C3—C2	119.67 (17)	C13—C12—H12	121.2
C4—C3—H3	120.2	C11—C12—H12	121.2
C2—C3—H3	120.2	F2—C13—C12	119.94 (19)
C3—C4—C5	121.01 (18)	F2—C13—C14	119.38 (19)
C3—C4—H4	119.5	C12—C13—C14	120.68 (18)
C5—C4—H4	119.5	F3—C14—C15	120.48 (19)
C4—C5—C6	121.01 (17)	F3—C14—C13	118.68 (18)
C4—C5—H5	119.5	C15—C14—C13	120.83 (19)
C6—C5—H5	119.5	C14—C15—C10	120.32 (18)
C5—C6—C1	116.76 (16)	C14—C15—H15	119.8
C5—C6—C7	121.31 (16)	C10—C15—H15	119.8
C1—C6—C7	121.89 (16)	C7—C16—C17	125.05 (17)
C16—C7—C6	119.77 (16)	C7—C16—H16	117.5
C16—C7—C8	120.87 (16)	C17—C16—H16	117.5
C6—C7—C8	119.33 (15)	O4—C17—O3	122.95 (17)
N1—C8—C7	115.47 (15)	O4—C17—C16	124.81 (16)
N1—C8—H8A	108.4	O3—C17—C16	112.24 (16)
C7—C8—H8A	108.4		
			
O1—S1—N1—C8	−49.50 (15)	S1—N1—C8—C7	−52.72 (19)
O2—S1—N1—C8	179.16 (13)	C16—C7—C8—N1	−173.12 (17)
C1—S1—N1—C8	64.22 (14)	C6—C7—C8—N1	9.0 (2)
O1—S1—N1—C9	177.10 (12)	C8—N1—C9—C10	138.74 (16)
O2—S1—N1—C9	45.76 (15)	S1—N1—C9—C10	−90.06 (17)
C1—S1—N1—C9	−69.18 (14)	N1—C9—C10—C11	140.18 (18)
O1—S1—C1—C2	−110.27 (17)	N1—C9—C10—C15	−44.1 (2)
O2—S1—C1—C2	23.12 (19)	C15—C10—C11—F1	179.92 (17)
N1—S1—C1—C2	137.38 (16)	C9—C10—C11—F1	−4.2 (3)
O1—S1—C1—C6	71.95 (16)	C15—C10—C11—C12	−1.0 (3)
O2—S1—C1—C6	−154.67 (14)	C9—C10—C11—C12	174.89 (18)
N1—S1—C1—C6	−40.41 (16)	F1—C11—C12—C13	178.87 (16)
C6—C1—C2—C3	−2.7 (3)	C10—C11—C12—C13	−0.2 (3)
S1—C1—C2—C3	179.63 (15)	C11—C12—C13—F2	−178.12 (17)
C1—C2—C3—C4	−0.3 (3)	C11—C12—C13—C14	1.3 (3)
C2—C3—C4—C5	3.0 (3)	F2—C13—C14—F3	−0.8 (3)
C3—C4—C5—C6	−2.7 (3)	C12—C13—C14—F3	179.76 (19)
C4—C5—C6—C1	−0.3 (3)	F2—C13—C14—C15	178.26 (19)
C4—C5—C6—C7	177.32 (17)	C12—C13—C14—C15	−1.1 (3)
C2—C1—C6—C5	3.0 (3)	F3—C14—C15—C10	178.94 (19)
S1—C1—C6—C5	−179.31 (13)	C13—C14—C15—C10	−0.1 (3)
C2—C1—C6—C7	−174.62 (17)	C11—C10—C15—C14	1.2 (3)
S1—C1—C6—C7	3.1 (2)	C9—C10—C15—C14	−174.72 (19)
C5—C6—C7—C16	21.0 (3)	C6—C7—C16—C17	−178.59 (16)
C1—C6—C7—C16	−161.52 (18)	C8—C7—C16—C17	3.5 (3)
C5—C6—C7—C8	−161.05 (17)	C7—C16—C17—O4	−4.6 (3)
C1—C6—C7—C8	16.4 (3)	C7—C16—C17—O3	176.17 (17)
C9—N1—C8—C7	79.7 (2)		

### Hydrogen-bond geometry (Å, º)

**Table:**

*D*—H···*A*	*D*—H	H···*A*	*D*···*A*	*D*—H···*A*
O3—H3*O*···O4^i^	0.95 (3)	1.71 (3)	2.6454 (19)	170 (3)
C12—H12···F3^ii^	0.95	2.50	3.448 (2)	178
C15—H15···F1^iii^	0.95	2.48	3.430 (2)	179
C5—H5···F2^iv^	0.95	2.49	3.269 (2)	140

Symmetry codes: (i) −*x*, −*y*, −*z*; (ii) *x*+1, *y*, *z*; (iii) *x*−1, *y*, *z*; (iv) *x*, *y*−1, *z*.
